# Exercise intensity in cancer survivors: a matter of the heart

**DOI:** 10.1186/s40959-017-0022-x

**Published:** 2017-03-13

**Authors:** Stephanie M. Smith, Joseph R. Carver

**Affiliations:** 1 Penn Medicine, University of Pennsylvania, Philadelphia, USA; 2 Abramson Cancer Center, University of Pennsylvania, Philadelphia, USA

A routine of regular aerobic exercise has been shown to have non-pharmacological benefits for the prevention and treatment of many potential side effects from chemotherapy. This includes the whole spectrum of cancer care from diagnosis through treatment and long-term survivorship. The case report by Korn et al that accompanies this editorial describes an adolescent leukemia survivor with anthracycline-induced cardiomyopathy who was able to train and compete in a triathlon. This is an important contribution that demonstrates to an extreme the physical capabilities of cancer survivors.

The role of exercise in the prevention and treatment of anthracycline cardiotoxicity remains controversial. In animal models, beneficial effects of exercise include preservation of left ventricular systolic and diastolic function by modulating heart rate and contractility through (a) an increase in antioxidant activity with reduction of reactive oxygen species release and increase in heat shock proteins; (b) a decrease in proapoptotic signaling; (c) a decrease in myocyte turnover through suppression of proapoptotic protein synthesis; and (d) changes in energy metabolism [1]. In humans, the data aremore limited. However, a case series of five long-term survivors of childhood cancer with subclinical anthracycline-induced cardiomyopathy demonstrated feasibility of a 12-week home aerobic and strength training program, and similar to the case report by Korn et al, survivors showed an improvement in exercise capacity and cardiopulmonary fitness (VO2 and O2 pulse). Importantly, these survivors were 25–30 years from cancer diagnosis and treatment completion, had an LVEF 40–50% at baseline, and did not experience any cardiac events or complications over the 12-week exercise program [2].

In the patient not exposed to potentially cardiotoxic chemotherapy, long-term excessive endurance exercise may induce pathologic structural remodeling of the heart – training and participation in “extreme” activities (marathons, triathlons) can increase pre-load and induce atrial and ventricular volume overload with liberation of cardiac troponins, and with continued training, may induce structural changes -- patchy areas of myocardial fibrosis, LV dilatation/hypertrophy and increase in myocardial mass [3].

This leads to a word of caution related to the intensity of exercise described in this case report. Vigorous activity with potential acute increases in catecholamines and increased energy demands can increase the risk of sudden death and acute myocardial infarction, especially in patients with underlying structural heart disease [4]. Patients with prior anthracycline exposure may have latent mitochondrial and myocardial dysfunction that reduces cardiac reserve and could magnify that risk, and additionally, the stress of exercise without adequate reserve may lead to clinical decomposition and overt heart failure.

Risk of sudden cardiac death has also been described in cancer survivors with cancer therapy-related cardiac dysfunction. In the general population, sudden cardiac death in young athletes <35 years of age is typically due to inherited conditions (structural pathology, arrhythmias) and in older athletes >35 years of age is typically due to coronary artery disease (CAD) [5]. Childhood cancer survivors are at risk for both – structural cardiomyopathy from direct toxic effects of chemotherapy or radiation therapy, as well as accelerated CAD, particularly if they received concurrent radiation therapy to the left chest in addition to cardiotoxic chemotherapy. The role for screening exercise testing in long-term childhood cancer survivors is not well delineated, but may be an important consideration before embarking on an intense exercise training program. In a study of screening echocardiography for more than 1800 adult survivors of childhood cancer treated with anthracycline chemotherapy (+/- radiation to the left chest), asymptomatic CAD was found in 69 patients (3.8%) and newly identified (not previously diagnosed) in 40 patients (2.2%), and valvular regurgitation or stenosis was found in 488 patients (28%), newly identified in 414 patients (23.8%) [6]. In pilot studies, screening CT coronary angiography has identified clinically significant CAD requiring percutaneous or surgical revascularization in asymptomatic survivors of Hodgkin lymphoma exposed to mediastinal radiation therapy; larger studies are needed to determine the utility of this approach for identifying CAD in cancer survivors at high risk [7–10].

The lack of consensus in the guidelines for exercise intensity among cancer survivors at high risk of cardiovascular late effects reflects the paucity of clinical studies in this area. The International Late Effects of Childhood Cancer Guideline Harmonization Group recommends cardiology consultation for cancer survivors with known therapy-related cardiomyopathy and for cancer survivors at high risk of cardiomyopathy prior to initiation of high-intensity exercise [11]. The American College of Sports Medicine recommends medical evaluation for adult cancer survivors with known cardiac conditions, whether treatment-related or not, to determine safe exercise limits before beginning an exercise program, but does not specifically advocate for assessment of asymptomatic survivors not known to have cardiac disease [12]. A study of adult cancer survivors who underwent cardiopulmonary exercise testing suggested that traditional American College of Sports Medicine cardiac risk stratification – which does not account for anthracycline or radiation therapy exposure – did not adequately describe exercise risk in this population of cancer survivors [13].

What is the answer? Maintaining balance and moderation is critical. A “little exercise” is good; “excessive exercise” may be detrimental. Therefore, our practice is to discourage extreme levels of exercise but recommend low-intensity endurance training and activities. Before prescribing a vigorous exercise program in patients previously exposed to anthracycline-based chemotherapy or radiation therapy to the left chest, patients should have:

1. Thorough screening history and physical exam that includes a current assessment of cardiac structure and function (echocardiogram and possibly cardiopulmonary exercise testing);
2. Education about possible warning “red-flag” symptoms;
3. Prescription for graded training with avoidance of high-risk activities, especially in the absence of adequate pre-training;
4. General recommendations about healthy lifestyle;
5. Regular supervision and follow-up with medical personnel
